# In Vitro Analysis of Outcome Differences Between Repairing and Replacing Broken Dental Restorations

**DOI:** 10.7759/cureus.56071

**Published:** 2024-03-13

**Authors:** Saraswati Sachan, Raktim De, Akshita Balivada, Soni Pandey, Neeraj K Tiwari, Supurna Franklin, Shivakumar Ganiga Channaiah, Shazia Siddiqui

**Affiliations:** 1 Department of Conservative Dentistry and Endodontics, Sardar Patel Post Graduate Institute of Dental and Medical Sciences, Lucknow, IND; 2 Department of Conservative Dentistry and Endodontics, Tooth Corner Dental Clinic and Endodontic Centre, Agartala, IND; 3 Department of Conservative Dentistry and Endodontics, RajaRajeswari Dental College & Hospital, Bangalore, IND; 4 Department of Conservative Dentistry and Endodontics, Radiant Dental Care, Lucknow, IND; 5 Department of Conservative Dentistry and Endodontics, Chandra Dental College & Hospital, Lucknow, IND; 6 Department of Conservative Dentistry and Endodontics, Saraswati Dental College and Hospital, Lucknow, IND; 7 Department of Oral Medicine and Radiology, People's College of Dental Sciences & Research Centre, Bhopal, IND; 8 Department of Conservative Dentistry and Endodontics, Career Post Graduate Institute of Dental Sciences & Hospital, Lucknow, IND

**Keywords:** glass ionomer cement, restorative materials, class ii cavities, repair, fracture, dental restorations, composite fillings

## Abstract

Objective

In light of several advancements and considerations in endodontic dentistry, there still remains a need to comprehensively evaluate the outcome disparities between repairing and replacing broken dental restorations. This study aims to compare the effectiveness of repairing dental restorations versus replacing them, focusing on how each method affects the structural strength and longevity of the restorations.

Methods

The study included 60 freshly removed human maxillary premolars. Initial processing involved rigorous washing, descaling, and polishing of the teeth. To ensure preservation, the specimens were stored in sterile, distilled water. To occlude the root canals, a self-hardening composite resin was used, and the roots were coated with two coats of clear nail polish to prevent moisture penetration. A 245 carbide bur attached to a high-speed dental handpiece with air and water spray cooling produced standardized Class II cavities on the occluso-proximal surfaces. Each cavity had a buccolingual breadth of 2 mm, an occluso-cervical length of 4 mm, and a gingival boundary that was 1 mm coronal to the cement-enamel junction. Following this preparation, the teeth were randomly separated into three groups (Group A, Group B, and Group C), each containing 20 teeth.

Results

Our analysis showed that teeth with entirely replaced restorations had a higher average fracture resistance than those with repaired restorations. However, the difference in fracture resistance between the repair and replacement groups for each type of material was not statistically significant.

Conclusion

Based on the findings, repairing a dental restoration can be a conservative and less invasive alternative to a full replacement without a significant compromise in the restoration’s ability to withstand fracture. Therefore, dental professionals might consider full restoration as a viable option, taking into account the need to preserve dental tissue as well as the restoration’s durability and structural integrity.

## Introduction

Dental research has continuously sought innovative approaches to address the challenges posed by dental diseases, particularly through reparative procedures. The integration of nanoscale engineering into dental biomaterials represents a significant milestone in this field. The advent of dental nanocomposites, enriched with nanoscale filler particles, signifies a transformative shift toward enhanced durability and aesthetic appeal in dental restorations [1]. Engineered with meticulously controlled particle size and increased filler content, these materials exhibit notable resistance to mechanical degradation, promising enduring restorative solutions. Traditionally, the predominant approach in dental restoration has favored the wholesale replacement of structurally compromised restorations. While conventional methods are characterized by simplicity, they entail the removal of not only the damaged restorative material but also additional dental tissue [2]. Such procedures may engender complications such as heightened dental sensitivity and the risk of pulpal injury, necessitating further therapeutic interventions. The advent of photoactivated resin-based composites has underscored the pivotal role of polymerization in determining the longevity and efficacy of restorative materials. The transition from monomeric to polymeric states is critical to optimizing the performance of these composites within the oral cavity. Moreover, the optical properties of these materials dictate the depth of light penetration, thereby influencing the efficacy of the polymerization process [3].

These optical properties (such as the translucency of the material, its refractive index, and color stability) are also very important for the mechanical strength [4] and biocompatibility [5] of the cured composite. Insufficient polymerization has been associated with a reduction in these crucial properties, which could adversely affect both the durability and operational effectiveness of the restoration [6]. Additionally, polymerization shrinkage-related stress is a constant problem. It has the potential to undermine the structural integrity of the material and exert considerable strain on the adhesive bond at the interface between the tooth and the restoration. Even with considerable improvements in the mechanical and physical characteristics of restorative materials, the success of dental restorations is not exclusively dependent on the materials’ properties. Clinical considerations such as case selection, operative isolation, abrasion resistance, cavity configuration, material handling, polymerization process, and subsequent finishing and polishing procedures play an instrumental role. Dislodgement or cracking are signs of neglect in any of these areas, which can cause the restoration to fail. In this study, we focus on determining the fracture toughness of various restorative materials, which is an important factor in evaluating their performance due to the constant compressive forces they must endure during chewing. Understanding how well a restorative material can withstand these forces is crucial. Therefore, our research is aimed at comparing the fracture resistance of dental restorations that have been repaired with those that have been entirely replaced.

## Materials and methods

Study setting

The research was carried out at the Department of Conservative Dentistry and Endodontics of the Sardar Patel Post Graduate Institute of Dental and Medical Sciences in Lucknow, India, with a collaborative partnership involving the Central Institute of Plastics Engineering & Technology (CIPET), also in Lucknow, India. The null hypothesis of the study posited that there would be no significant difference in fracture resistance between dental restorations repaired with specific materials and those entirely replaced.

Selection criteria for teeth

**Figure 1:**
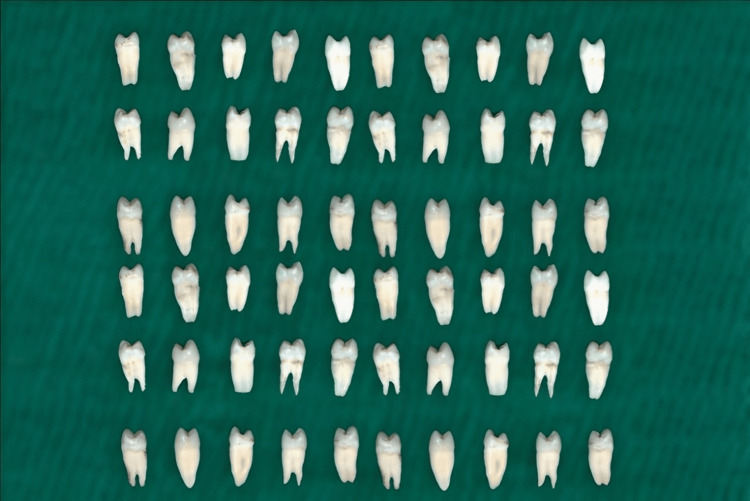
Samples before cavity preparation

Teeth that were devoid of caries, fissures, and developmental irregularities were considered for inclusion in this study. Teeth with unaltered crown architecture were also considered to be eligible for inclusion. In terms of the exclusion criteria, teeth that displayed signs of attrition, abrasion, and erosion or had undergone prior restorative procedures were considered ineligible for the study. After the application of these criteria, a total of 60 freshly extracted human maxillary premolars were selected for the study, as shown in Figure 1. Initial processing included meticulous cleaning, descaling, and smoothing of the teeth. The specimens were stored in sterilized, distilled water for preservation. To occlude the root canals, a self-hardening composite resin was utilized, and the roots were coated with two applications of clear nail polish to avert moisture penetration. A 245 carbide bur connected to a high-speed dental handpiece that used air and water spray cooling created standardized Class II cavities on the occluso-proximal surfaces, with the bur being preferred for its efficiency in cavity preparation and its ability to achieve precise and standardized cavity dimensions, which allows for effective removal of dental tissue while minimizing trauma to surrounding structures. The dimensional criteria for each cavity included a buccolingual breadth of 2 mm, an occluso-cervical length of 4 mm, and a gingival margin set at 1 mm coronal to the cementoenamel junction. Following this preparation, the teeth were randomly divided into three groups (Group A, Group B, and Group C), with each consisting of 20 teeth.

Restoration protocol

The study incorporated three distinct restorative materials: Filtek Supreme (a nanofill composite), Filtek Bulkfill (a bulkfill composite), and HI DENSE (a silver-reinforced glass ionomer cement).

Group A (20 Samples)

A universal dental adhesive (3M ESPE Adper Single Bond Universal Agent, 3M Company, Saint Paul, Minnesota, USA) was applied to the cavity surfaces and cured with light for 10 seconds according to the manufacturer’s specifications. Filtek Supreme was used to fill the cavities, ensuring complete coverage. The material was then molded to replicate the original tooth’s morphology and subjected to further light curing for 20 seconds.

Group B (20 Samples)

An identical bonding and curing regimen to Group A was followed. Filtek Bulkfill composite was placed and adapted to the cavity, ensuring it conformed to all surfaces and margins, followed by light curing for 20 seconds as instructed.

Group C (20 Samples)

A blend of two measures of powder and two drops of liquid from the glass ionomer cement (HI DENSE) was mixed. This mixture was then transferred into the cavities, packed firmly to ensure coverage of all internal aspects and margins, and then shaped to mirror the tooth’s natural anatomy.

**Figure 2:**
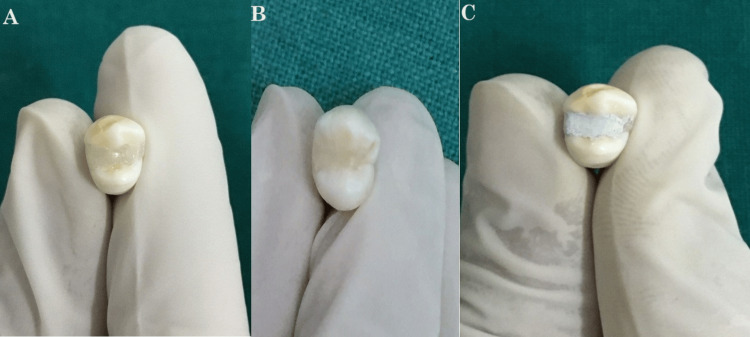
Restoration process representation: (A) Nanofill, (B) Bulkfill, and (C) Cermet

The restoration process using the three different compounds is shown in Figure 2.

**Figure 3:**
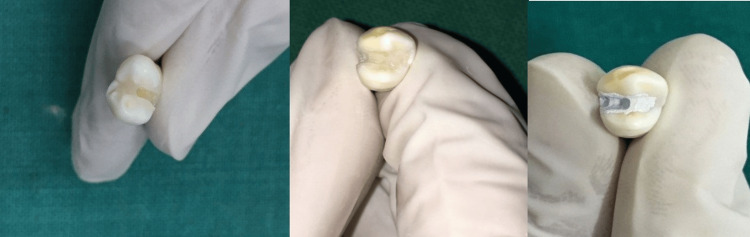
Samples after repair cavity preparation

On the other hand, the reparation process is elucidated in Figure 3. The finishing and polishing process followed the shaping of the restorations, and any excess material was carefully removed. The surfaces were refined to achieve a smooth and uniform texture. The polishing procedure involved the use of abrasive materials and polishing agents to refine the surface of the restorations further. This step aimed to enhance the aesthetic appearance of the restorations while minimizing surface irregularities that could potentially harbor plaque and bacteria. Upon completion of either setting or curing, depending on the material used, the restorations were finished, polished, and then stored in distilled water at a temperature of 37°C for 30 days.

Group protocols

A minimal Class II cavity recreation, measuring 1 mm in width and 2 mm in depth, was executed along the margins of the existing restorations to simulate a defect. This was performed using a high-speed handpiece with diamond abrasive points under air-water cooling. The dimensions of these secondary cavity preparations were verified with a Williams probe for consistency. The A1 subgroup was repaired with Filtek Supreme, B1 with Filtek Bulkfill, and C1 with HI DENSE, according to the respective materials initially used for restoration. For the restorations designated for complete replacement, the existing material was meticulously extracted utilizing a high-speed handpiece equipped with air/water cooling and a #245 carbide bur. To ensure that all subsequent restorations matched in terms of size, a periodontal probe was used to measure the dimensions of the cavities consistently. The A2 subgroup had their restorations replaced with Filtek Supreme, the B2 subgroup with Filtek Bulkfill, and the C2 subgroup with HI Dense, respectively. Each of the 60 specimens underwent embedding in a self-curing acrylic resin block. This process was carried out with precision to align the occlusal surfaces of the teeth parallel to the surface of the resin block. Post-embedding, the specimens were submerged in distilled water and maintained at a temperature of 37°C for 24 hours. This step was crucial as it aimed to replicate thermal conditions similar to those in the human oral environment.

Fracture resistance testing

The fracture resistance of the prepared samples was assessed using a universal testing machine (Instron, Norwood, Massachusetts, USA). Each specimen was secured within a holder slot attached to the lower arm of the testing apparatus. A metal indenter connected to the upper arm of the machine was employed to apply a vertical force at a consistent crosshead speed of 1 mm per minute. The force was applied until the sample failed, and the maximum load at the point of failure was recorded in MPa. Table 1 shows the obtained values across different groups across the 10 times that the force application was performed.

**Table 1:** Fracture resistance values observed across the different groups

Nanofill composite (Group A)		Bulkfill composite (Group B)		Cement (Group C)	
Repair group (A1)	Replaced group (A2)	Repair group (B1)	Replaced group (B2)	Repair group (C1)	Replaced group (C2)
11.5 MPa	12.5 MPa	9.87 MPa	6.67 MPa	8.76 MPa	3.89 MPa
9.13 MPa	10.13 MPa	8.67 MPa	9.67 MPa	1.89 MPa	8.67 MPa
7.87 MPa	6.57 MPa	11.36 MPa	13.36 MPa	5.32 MPa	3.23 MPa
11.7 MPa	13.7 MPa	9.40 MPa	7.40 MPa	2.87 MPa	2.67 MPa
16.7 MPa	11.9 MPa	7.86 MPa	8.89 MPa	1.89 MPa	3.45 MPa
8.98 MPa	15.7 MPa	11.89 MPa	11.63 MPa	4.89 MPa	4.50 MPa
9.67 MPa	8.65 MPa	6.87 MPa	9.92 MPa	2.50 MPa	3.56 MPa
15.5 MPa	14.7 MPa	7.67 MPa	12.84 MPa	3.54 MPa	2.93 MPa
11.2 MPa	13.2 MPa	12.85 MPa	9.32 MPa	2.76 MPa	1.89 MPa
9.23 MPa	10.23 MPa	7.63 MPa	6.83 MPa	3.23 MPa	5.47 MPa

**Figure 4:**
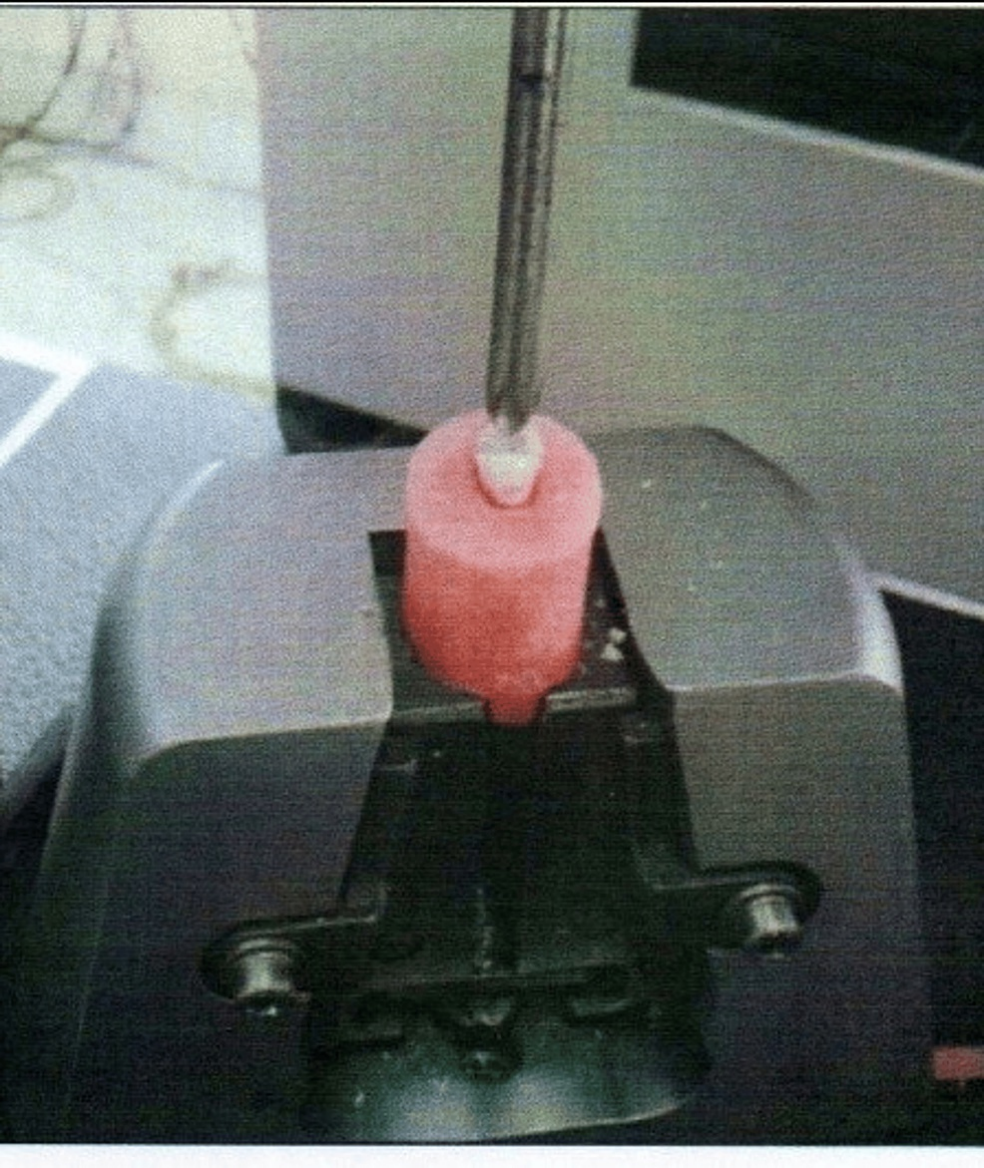
Fracture testing of the restored tooth

Figure 4 shows the fracture testing process for the tooth after embedding it in an acrylic resin block.

Statistical analysis

Data analysis was conducted using SPSS for Windows, Version 15.0 (Released 2006; SPSS Inc.: Chicago, Illinois, USA). The results were presented in terms of frequency (number) and descriptive statistics (mean ± SD). ANOVA and Tukey’s honestly significant difference (HSD) tests were performed to assess intragroup as well as intergroup comparisons. The statistical software facilitated the comprehensive analysis of the fracture resistance data among the different groups and subgroups.

## Results

Table 2 shows the fracture resistance analysis of the repaired specimens across different parameters. In Group A1, consisting of 10 specimens, the fracture resistance values ranged from a minimum of 7.87 MPa to a maximum of 16.70 MPa, with an average fracture resistance of approximately 11.15 MPa and a SD of 2.90 MPa. Similarly, Group B1, also comprising 10 specimens, exhibited a fracture resistance range from 6.87 MPa to 12.85 MPa, with a mean fracture resistance of approximately 9.41 MPa and a SD of 2.04 MPa. Conversely, Group C1, with 10 specimens, displayed the lowest fracture resistance values, ranging from 1.89 MPa to 8.76 MPa, resulting in an average fracture resistance of about 3.77 MPa and a SD of 2.09 MPa. Upon interpretation of the data, it is evident that Group A1, repaired with Filtek Supreme, demonstrated the highest average fracture resistance among the three groups, followed by Group B1, repaired with Filtek Bulkfill. Group C1, repaired with high-density cement, exhibited the lowest average fracture resistance. The significant p-value indicates that there were statistically significant differences in fracture resistance among the repaired specimens in the three groups. The ANOVA conducted on the data showed a significant difference in fracture resistance between the groups, as evidenced by a sum of squares value of 297.91 for between-group variability and a notably high F-value of 26.357. Among the repaired specimens, Group A1, repaired with Filtek Supreme, exhibited the highest fracture resistance compared to Groups B1 and C1. The average fracture resistance for Group A1 was approximately 11.15 MPa, which was higher than the average fracture resistance values observed in Groups B1 and C1.

**Table 2:** Tabular representation of the fracture resistance analysis of the repaired specimens across different parameters A p-value of 0.001 was considered significant.

Group	No. of specimens	Min.	Max.	Mean	SD	p-value
Group A1	10	7.87	16.7	11.15	2.9	<0.001
Group B1	10	6.87	12.85	9.41	2.04	
Group C1	10	1.89	8.76	3.77	2.09	
Total	30	1.89	16.7	8.11	3.94	

Table 3 provides a focused comparison of the mean differences in fracture resistance between the groups using Tukey’s HSD test. The comparison between Group A1 and Group B1 had a mean difference of 1.74 MPa with a standard error of 1.06 MPa, which was not statistically significant, as suggested by a p-value of 0.248. Conversely, the mean fracture resistance was significantly different when comparing Group A1 to Group C1, with a mean difference of 7.38 MPa and a p-value of less than 0.001. This significant difference was also observed between Group B1 and Group C1, where the mean difference of 5.64 MPa was also supported by a p-value of less than 0.001. These results indicate that Groups A1 and B1 had similar fracture resistances, whereas both had significantly higher fracture resistances compared to Group C1.

**Table 3:** Intergroup comparison of fracture resistance of repaired specimens (Tukey HSD test) A p-value of 0.001 was considered significant. HSD, honestly significant difference

Group	Mean difference	SE	p-value
Group A1 vs. Group B1	1.74	1.06	0.248
Group A1 vs. Group C1	7.38	1.06	<0.001
Group B1 vs. Group C1	5.64	1.06	<0.001

Table 4 shows the fracture resistance analysis of the replaced specimens across different parameters. In Group A2, the fracture resistance ranged from 6.57 MPa to 15.70 MPa, with an average resistance of 11.73 MPa and a SD of 2.83 MPa, reflecting a moderate spread of data points. Group B2 reported fracture resistance numbers spanning from 6.67 MPa to 13.36 MPa, with a mean value of 9.65 MPa and a SD of 2.37 MPa, indicating a slightly tighter distribution compared to Group A2. Group C2 displayed the lowest fracture resistance values, ranging from 1.89 MPa to 8.67 MPa, with an average of 4.03 MPa and a SD of 1.91 MPa, suggesting the least variation among its specimens. The overall range of fracture resistance across all replaced specimens was from 1.89 MPa to 15.70 MPa, with a mean of 8.47 MPa and a SD of 4.04 MPa, indicating a broad dispersion of fracture resistance values. The ANOVA reflected significant differences in fracture resistance among the three groups. With a sum of squares for the groups at 317.63 and a mean square of 158.82, the F-value reached 27.278, and the p-value was less than 0.001, signifying a statistically significant difference between the groups that was unlikely to have occurred by chance.

**Table 4:** Tabular representation of the fracture resistance analysis of the replaced specimens across different parameters A p-value of 0.001 was considered significant.

Group	No. of specimens	Min.	Max.	Mean	SD	p-value
Group A2	10	6.57	15.7	11.73	2.83	<0.001
Group B2	10	6.67	13.36	9.65	2.37	
Group C2	10	1.89	8.67	4.03	1.91	
Total	30	1.89	15.7	8.47	4.04	

Further analysis of the replaced specimens is presented in Table 5, which outlines the mean differences in fracture resistance between the groups as determined by the Tukey HSD test. The comparison between Group A2 and Group B2 revealed a mean difference of 2.08 MPa with a standard error of 1.07 MPa, a difference that was not statistically significant given the p-value of 0.149. In contrast, the difference between Group A2 and Group C2 was substantial, with a mean difference of 7.70 MPa and a p-value of less than 0.001, indicating a significant disparity in fracture resistance. Similarly, the comparison between Group B2 and Group C2 yielded a mean difference of 5.63 MPa with a p-value of less than 0.001, further illustrating a significant difference in fracture resistance.

**Table 5:** Intergroup comparison of fracture resistance of repaired specimens (Tukey HSD test) A p-value of 0.001 was considered significant. HSD, honestly significant difference

Group	Mean difference	SE	p-value
Group A2 vs. Group B2	2.08	1.07	0.149
Group A2 vs. Group C2	7.7	1.07	<0.001
Group B2 vs. Group C2	5.63	1.07	<0.001

**Figure 5:**
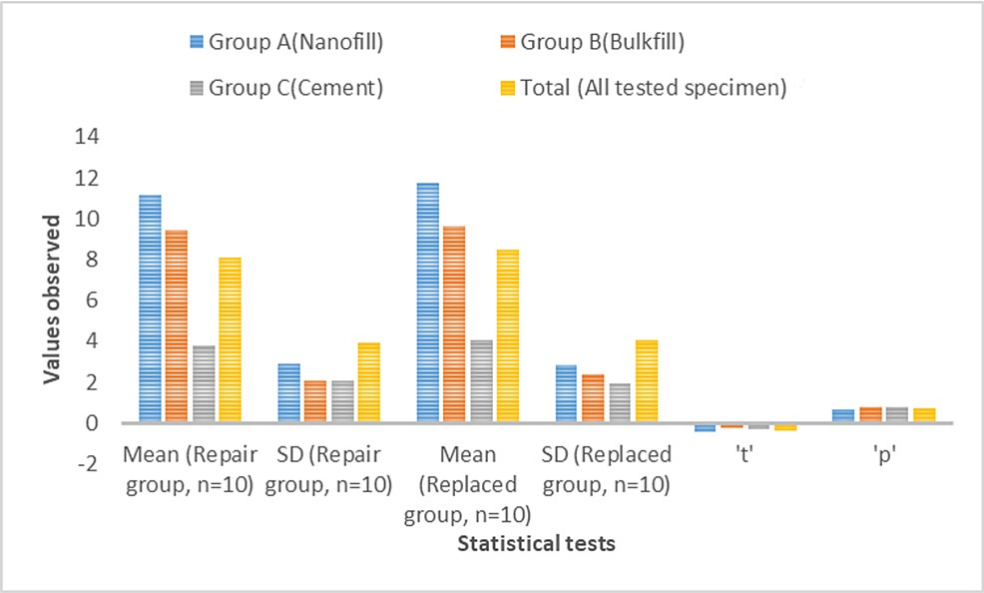
Overall intergroup comparison of fracture resistance of repaired and replaced specimens A p-value of 0.001 was considered significant.

Figure 5 shows the difference in mean fracture resistance between repaired specimens (11.15 + 2.90 MPa) and replaced specimens (11.73 + 2.83 MPa) in Group A, which was not found to be statistically significant (p = 0.656). The difference in mean fracture resistance between repaired specimens (9.41 + 2.04 MPa) and replaced specimens (9.65 + 2.37 MPa) in Group B was not found to be statistically significant (p = 0.806). The difference in mean fracture resistance between repaired specimens (3.77 + 2.09 MPa) and replaced specimens (4.03 + 1.91 MPa) in Group C was not found to be statistically significant (p = 0.774). The difference in mean fracture resistance between repaired specimens (8.11 + 3.94 MPa) and replaced specimens (8.47 + 4.04 MPa) of all the specimens was not found to be statistically significant (p = 0.726). The mean fracture resistance of replaced specimens was found to be higher than that of repaired specimens for each of the groups, but differences were not found to be statistically significant.

## Discussion

Our findings revealed that the assumed null hypothesis was not upheld. The results indicated notable discrepancies in fracture resistance between restorations repaired with different materials and those entirely replaced. The variations observed across the different groups and subgroups underscored the impact of restorative materials and procedures on the fracture resistance of dental restorations. Specifically, the fracture resistance values obtained from the repair and replacement groups exhibited significant differences, indicating that the choice of restorative material and procedure significantly influenced the structural integrity and durability of the restorations. These findings highlighted the importance of careful consideration and selection of materials and techniques in dental restoration procedures to optimize fracture resistance and long-term clinical outcomes. Moreover, the study highlighted differences in fracture resistance between repaired and replaced restorations. Repaired restorations generally exhibit higher fracture resistance compared to replaced ones. This indicates that repairing broken dental restorations can potentially restore their structural integrity more effectively than replacing them entirely. The statistical analyses further supported these findings by revealing significant differences in fracture resistance among the various groups studied, underscoring the importance of considering both material selection and repair/replacement strategies in clinical practice.

Contemporary dental practices are increasingly adopting techniques that conserve tooth structure and employ advanced restorative materials like resin composites. These composites are made to bond well to tooth structures [7,8] and are still preferred even though they tend to shrink during polymerization, which can put stress on the tooth restoration interface and cause microleakage. To minimize this effect, composites with higher filler content, such as nanofill and bulkfill composites, have been introduced [9]. The addition of silver has also improved the properties of glass ionomer cement for use in clinical settings. When the marginal ridge is removed during Class II cavity preparation, which raises the risk of microfractures when chewing, this study looks at the mechanical integrity of teeth afterward. Removal of the marginal ridge decreases the tooth’s structural resistance significantly, often reported to be around 60%, with one study noting a 46% decrease in resistance [10]. The difficulty in isolating and accessing the proximal areas of posterior teeth further heightens the possibility of fracture and restoration failure. It is recognized that wider occlusal isthmuses in Class II cavities can increase fracture susceptibility [11].

The maxillary premolars were chosen for this study because they have good anatomical and functional qualities that make them good models for testing how resistant restorative materials are to breaking. The particular shape and crown-to-root ratio of these teeth are thought to confer greater fracture resistance. In contrast, the angle of cusps in mandibular premolars and their position in the dental arch subject them to different forces, which may increase their fracture risk [10]. Composite resin restorations can fail for various reasons. One common issue is aesthetic degradation, such as discoloration, which impacts patient satisfaction and may require restoration replacement. Loss of marginal integrity can also cause discomfort or pain, necessitating another reason for the replacement of restorations [12]. Current practice often involves the total replacement of failed restorations. However, the choice between replacing or repairing a restoration should consider the balance of pros and cons. The complete replacement carries the risk of removing additional healthy tooth structures and possibly damaging the pulp. On the other hand, repair is seen as a less invasive alternative that can be more favorable for patients [13].

In this investigation, we assessed the bond strength of restorative materials to dentin by measuring fracture strength, a reliable indicator of material performance under stress. The fracture resistance test was employed due to its straightforward approach to quantifying the bond strength of dental repair materials [14]. Our examination of the mechanical properties of dental restorative materials, especially under high masticatory load simulations [15], was conducted using a universal testing machine. We applied a uniaxial compressive force longitudinally to each tooth specimen at a displacement rate of 1 millimeter per minute, quantifying the materials’ response to axial stress. The data indicate that replacing the entire restorative material tends to improve fracture resistance, likely due to the uniformity and integrity of the new material. This was particularly evident in the group where the restorative material was entirely replaced, suggesting optimal fracture resistance characteristics inherent to the material in an undamaged condition. In the subset of specimens using glass ionomer cement (Group C), fractures predominantly occurred within the material itself. Analysis of the fracture surfaces showed that the cement maintained its adherence to the cavity walls, indicating a strong bond to dentin, which is a known benefit of glass ionomer cement due to its chemical adhesion properties and fluoride release.

Despite observing greater fracture resistance in restorations that were completely replaced, statistical analysis showed no significant difference when compared to the resistance of repaired restorations. This supports the strategy of repairing restorations rather than full replacement, aligning with a conservative treatment philosophy that emphasizes tooth preservation, cost-effectiveness, patient comfort, and the potential for extending the lifespan of the dental structure. Supporting these observations, Gordan et al. [16] examined alternatives to replacing defective amalgam restorations and recognized repair as a viable option. Javidi et al. [17] also found composite repair to be a favorable choice due to its less invasive nature and ability to extend the life of the restoration. Moncada et al. [18], in a clinical trial, found that non-replacement treatments for compromised Class I and II resin-based composite and amalgam restorations showed improved outcomes over three years, corroborating the results of our study. Recognizing the nuanced differences between clinical and experimental fracture conditions is paramount. Clinical settings encounter variable intraoral forces that are not perfectly mirrored by the static, uniaxial application of force in laboratory settings. Thus, additional studies are necessary to more accurately align in vitro findings with clinical performance. The experiment was designed to compare the fracture resistance of repaired versus completely replaced dental restorations. Standardized Class II cavities were prepared on 60 extracted human teeth, which were then divided into three groups of 20, with each group receiving a different restorative material.

The results indicated that completely replaced restorations possessed a higher average fracture resistance, yet the statistical insignificance of the difference from repaired restorations suggests a nuanced interpretation. Notwithstanding these insights, the in vitro nature of the study is a constraining factor due to the absence of complex oral biomechanics and the controlled nature of the applied forces, which differ from the multifarious forces in the oral cavity. The sample size of 60 teeth might be perceived as sufficient; however, it may not be statistically robust enough to identify subtle, albeit clinically pertinent, distinctions between the repair and replacement strategies. The study’s design, which grouped the samples according to the restorative material, did not take into account the extensive variety of materials and techniques prevalent in modern dental practice, which could limit the broader applicability of the findings. Additionally, the immediate fracture resistance data used to infer the longevity of the restorations does not account for potential long-term wear and degradation. The absence of an extended follow-up period restricts the study’s scope to initial laboratory outcomes and prevents any long-term prognostic conclusions about the comparative success of repaired versus replaced restorations. Therefore, while the study contributes valuable initial data, its conclusions must be cautiously considered in light of these limitations. It underscores the necessity for ongoing clinical trials to validate the laboratory findings within the context of the authentic oral environment.

## Conclusions

Our findings reveal a clear hierarchy in fracture resistance: nanofill composite > bulkfill composite > cermet material. While completely replacing restorations showed slightly higher fracture resistance than repair, this difference was not statistically significant. Thus, while replacement may offer marginally higher resistance, repair remains a viable, less invasive option that effectively prolongs restoration life. Therefore, our study advocates for considering repair as a preferred choice when clinically appropriate, underscoring its equivalence to replacement in maintaining tooth restoration integrity and emphasizing its conservative and effective nature. In essence, our assessments highlight the fact that, with respect to fracture resistance, repaired restorations were essentially equivalent to replaced restorations. This reinforces the notion that repairs are not only a conservative approach but also an effective one for maintaining the integrity of the tooth restoration complex, highlighting the clinical significance of our assessments.
